# Stratification to predict the response to antioxidant

**DOI:** 10.5935/0103-507X.20200016

**Published:** 2020

**Authors:** Cristiane Ritter, Larissa Constantino, Monique Michels, Renata Casagrande Gonçalves, Cassiana Fraga, Danusa Damásio, Felipe Dal-Pizzol

**Affiliations:** 1 Postgraduate Program in Health Sciences, Laboratory of Experimental Pathophysiology, Universidade do Extremo Sul Catarinense - Criciúma (SC), Brazil.; 2 Research Center, Hospital São José - Criciúma (SC), Brazil.

**Keywords:** Interleukin-6, Antioxidants, Sepsis, Inflammation mediators, Models, animal, Rats, Interleucina-6, Antioxidantes, Sepse, Mediadores da inflamação, Modelos animais, Ratos

## Abstract

**Objective:**

To examine the effectiveness of stratification to identify and target antioxidant therapy for animal models of lethal sepsis and in patients who develop sustained hypotension.

**Methods:**

Rats were subjected to sepsis induced by cecal ligation and puncture. Animals were divided into two groups: those with high and low plasma levels of interleukin-6. Following stratification, N-acetylcysteine plus deferoxamine or saline was administered to animals starting 3 and 12 hours after surgery. N-Acetylcysteine plus deferoxamine or placebo was administered within 12 hours of meeting the inclusion criteria in hypotensive patients.

**Results:**

N-Acetylcysteine plus deferoxamine increased survival in the cecal ligation and puncture model when administered 3 and 12 hours after sepsis induction. When dividing animals that received antioxidants using plasma interleukin-6 levels, the protective effect was observed only in those animals with high IL-6 levels. The antioxidant effect of N-acetylcysteine + deferoxamine was similar in the two groups, but a significant decrease in plasma interleukin-6 levels was observed in the high-interleukin-6-level group. Compared with patients treated with antioxidants in the low-interleukin-6 subgroup, those in the high-interleukin-6 subgroup had a lower incidence of acute kidney injury but were not different in terms of acute kidney injury severity or intensive care unit mortality.

**Conclusion:**

Targeting antioxidant therapy to a high inflammatory phenotype would select a responsive population.

## Abstract (pt)

**Objetivo:**

Avaliar a efetividade da estratificação para identificar e escolher alvos para terapia antioxidante em um modelo de sepse letal em animais e pacientes que desenvolveram hipotensão prolongada.

**Métodos:**

Submeteu-se um grupo de ratos à sepse induzida por ligadura e punção do ceco. Os animais foram divididos em dois grupos: os com níveis plasmáticos altos e os com níveis plasmáticos baixos de interleucina-6. Após a estratificação, administrou-se aos animais N-acetilcisteína mais desferroxamina ou soro fisiológico a partir de 3 e 12 horas após a cirurgia. Em pacientes hipotensos, N-acetilcisteína mais desferroxamina ou placebo foram administrados dentro de 12 horas após o cumprimento dos critérios para inclusão.

**Resultados:**

O uso de N-acetilcisteína mais desferroxamina aumentou a sobrevivência no modelo com ligadura mais punção do ceco quando a administração ocorreu 3 e 12 horas após indução da sepse. Ao utilizar os níveis de interleucina-6 para separar os animais que receberam antioxidantes, o efeito protetor só foi observado nos animais que tinham níveis elevados de interleucina-6. O efeito antioxidante de N-acetilcisteína mais desferroxamina foi similar nos dois grupos, porém observou-se diminuição significante dos níveis plasmáticos de interleucina-6 no grupo que apresentava elevado nível de interleucina-6. Em comparação com pacientes tratados com antioxidantes no subgrupo que tinha baixos níveis plasmáticos de interleucina-6, aqueles que tinham níveis elevados de interleucina-6 tiveram menor incidência de lesão renal aguda, porém não foram diferentes em termos de severidade da lesão renal aguda ou da mortalidade na unidade de terapia intensiva.

**Conclusão:**

Direcionar a terapia antioxidante para um elevado fenótipo inflamatório selecionaria uma população responsiva.

## INTRODUCTION

Sepsis remains a health challenge, with millions of cases every year, high rates of mortality and morbidity, and impaired quality of life among survivors.^(1)^ Several strategies have been proposed to improve mortality in sepsis, one of them being the use of biomarkers to predict the response to treatment.^(2)^ Using different biomarkers, from cytokines to echocardiographic findings, it is possible to develop an approach that allows accurate mortality prediction in animal models of sepsis.^(3-6)^In this context, an individualized evaluation of the animals’ immunoinflammatory status/risk of death based on plasma levels of interleukin (IL)-6 was proposed that allows targeted immunomodulation and improved survival only in the appropriate group.^(7,8)^This same approach was used to retrospectively determine the efficacy of *anti*-*tumor necrosis factor* (anti-TNF) therapy only in a subgroup of septic patients presenting with high IL-6 levels.^(9)^

The use of antioxidants in sepsis is still a matter of debate.^(10)^ Despite their general effectiveness in animal models ,^(11-14)^ results on the use of antioxidants in humans are still inconclusive.^(14-16)^We previously demonstrated the advantage of the combined use of different antioxidants, N-acetylcysteine (NAC) plus deferoxamine (DFX), compared to their use alone in different animal models of critical illness.^(11-13)^When NAC and DFX were administered to humans, they decreased plasma levels of oxidative damage and inflammatory parameters,^(17)^ but they did not decrease the incidence of acute kidney injury (AKI).^(18)^In this context, the use of biomarkers could improve subgroup selection to the use of NAC plus DFX.

To verify this approach in a scenario of targeted treatment, we hypothesized that it is possible to stratify both septic animals and critically ill patients using plasma IL-6 levels to identify responders to antioxidant therapy.

The aim this study was to examine the effectiveness of stratification to identify and target antioxidant therapy for animal models of lethal sepsis and in patients who develop sustained hypotension.

## METHODS

### Animals and study design

Two-month-old adult male *Wistar* rats (250 - 300g) were used. Animals were housed in groups of five with free access to food and water and were maintained on a 12-h light-dark cycle (lights on 7:00 am) at a temperature of 22ºC ± 1ºC. All experimental procedures were carried out in accordance with the National Institutes of Health Guidelines, and approval was obtained from the institutional ethics committee of the *Universidade do Extremo Sul Catarinense* under protocol number 018/2019-1.

### Cecal ligation puncture model

Male Wistar rats were subjected to the cecal ligation puncture (CLP) procedure as previously described,^(19)^with minor modifications.^(20)^ Briefly, under aseptic conditions, a 3cm midline laparotomy was performed to expose the cecum. The cecum was tightly ligated with a 3.0 silk suture at its base, below the ileocecal valve, and was perforated once with a 14-gauge needle. The cecum was then gently squeezed to extrude a small amount of feces from the perforation site. Animals were resuscitated with regular saline (30mL/kg) subcutaneously immediately after and 12 hours after CLP. To minimize variability between different experiments, the CLP procedure was always performed by the same investigator. All animals were returned to their cages with free access to food and water.

### Experimental protocols

Animals were studied in two different protocols. In the first protocol (n = 90), sepsis was induced, and three hours after blood was collected from the caudal vein to determine IL-6 levels, treatment was started. In the second protocol (n = 90), sepsis was induced, blood was collected three hours later, and treatment was started 12 hours after CLP. At both times, animals were randomized to receive either antibiotics (ceftriaxone at 30mg/kg, every 12 hours and clindamycin 25mg/kg every 8 hours starting 3 hours or 12 hours after CLP) (antibiotics - ATB group) or antibiotics plus NAC (20mg/kg) every 6 hours plus DFX (20mg/kg once a day) (ATX group) for 3 consecutive days. To predict the response to ATX, animals were divided into (1) a high-IL-6 group (IL-6 ≥ 2000pg/mL) and (2) a low-IL-6 group (IL-6 < 2000pg/mL). These values of plasma IL-6 levels were based on previous studies^(21)^and were confirmed by pilot studies in our model. In these experiments, the mortality rate of the animals was recorded over a 5-day period.

In both protocols, blood was collected from the caudal vein 24 hours after CLP to determine plasma cytokine and oxidative damage parameter levels.

### Measurements

As an index of oxidative damage, the formation of thiobarbituric acid reactive species (TBARS) was used during an acid-heating reaction as previously described.^(22)^ Briefly, the samples were mixed with 1mL of 10% trichloroacetic acid and 1mL of 0.67% thiobarbituric acid and then heated in a boiling-water bath for 15 mins. Thiobarbituric acid reactive species were determined by the absorbance at 535nm using 1,1,3,3-tetramethoxypropane as an external standard. To minimize peroxidation during the assay procedure, butylated hydroxytoluene was added to the thiobarbituric acid (TBA) reagent mixture. The results are expressed as malondialdehyde equivalents per milligram of protein.

The oxidative damage to proteins was assessed by the determination of carbonyl groups based on the reaction with dinitrophenylhydrazine as previously described.^(23)^ Briefly, proteins were precipitated by the addition of 20% trichloroacetic acid and redissolved in dinitrophenylhydrazine, and the absorbance was read at 370nm.

Interleukin-6 was determined by ELISA with commercially available kits (R&D System) and expressed as pg/mL.

### Patients study

The trial was approved by the *Hospital São José* Institutional Review Board (CAAE - 0021.0.379.139-08).

To further determine the potential value of IL-6 in the prediction of the response to antioxidants, a subanalysis of a previously published study that determined the efficacy of NAC + DFX in the incidence of AKI in critically ill patients who developed sustained hypotension was performed.^(18)^ Briefly, patients were included if they had new onset of 30 consecutive minutes of hypotension, defined as a mean arterial blood pressure < 60mmHg that did not improve after fluid infusion or requirement for vasopressor medication (dopamine drip at 10µg/kg/min or norepinephrine, epinephrine, or vasopressin drip at any concentration). The first dose of NAC plus DFX, or placebo, had to be given within 12 hours of meeting the inclusion criteria. A loading dose of 50mg/kg NAC diluted in 250mL of 5% glucose was administered intravenously at an infusion rate of 62.5mL/h. After the loading dose, patients received a continuous infusion of NAC 100mg/kg/day diluted in 250mL of 5% glucose for two consecutive days. Deferoxamine was administered once at a dose of 1000mg dissolved in 250mL of 5% glucose at a rate of 3.75mL/kg/h. Blood was collected upon enrollment for the determination of IL-6 using commercial ELISA kits (R&D System). The median IL-6 level (949pg/mL) was used to stratify patients on the basis of high and low IL-6 levels.

### Statistical analysis

Oxidative damage and inflammatory parameters were expressed as the mean ± the standard deviation and compared by two-way ANOVA. In the survival experiments, the survival curves of the different treatment groups were compared using a log-rank test. In humans, a comparison between groups was performed using the chi-squared test. Statistical significance was defined as p *<* 0.05.

## RESULTS

**Figure 1:**
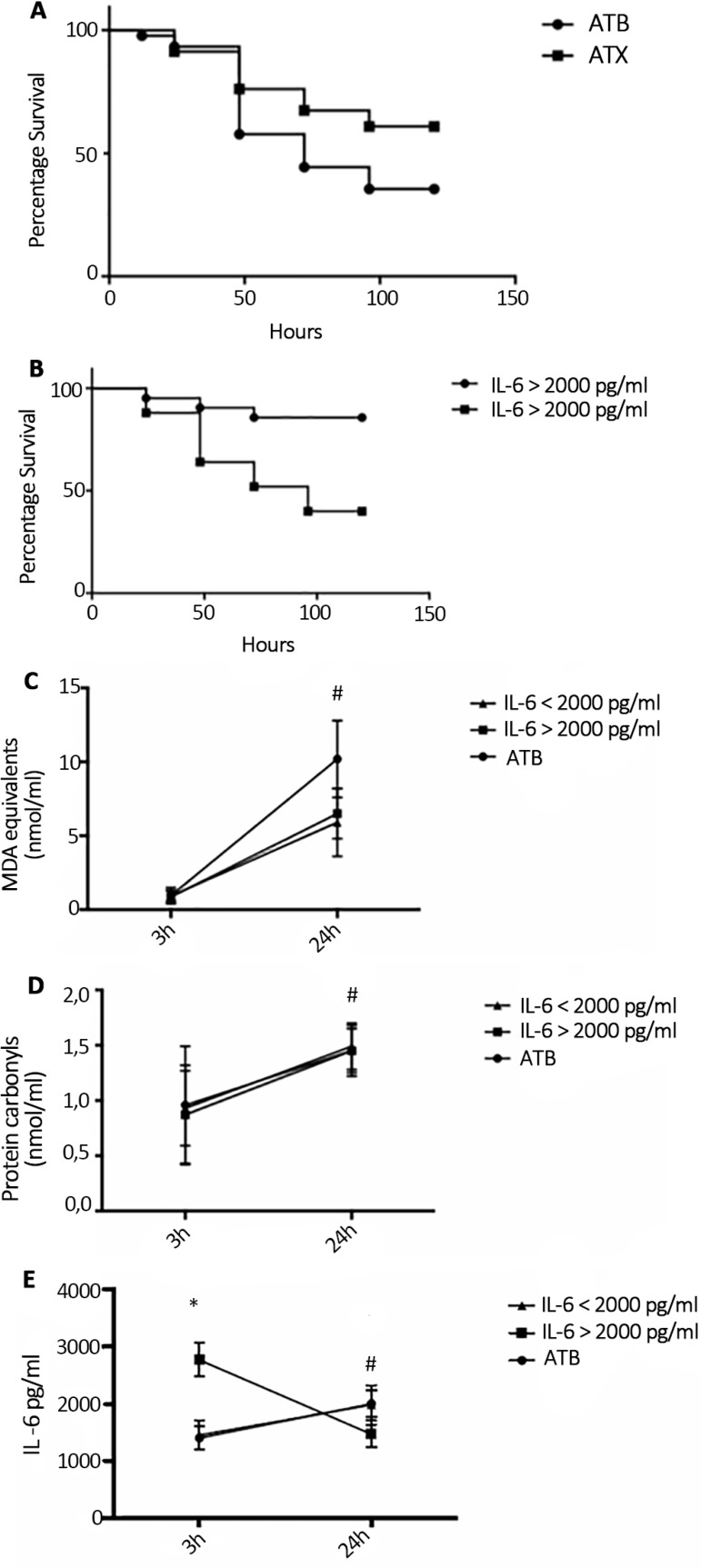
Effects of antioxidant administration three hours after sepsis induction in an animal model. (A) Mortality rate comparing the use of antibiotics with antibiotics plus antioxidants. (B) Mortality rate comparing the use of antibiotics plus antioxidants in animals in the high- and low-interleukin-6-level groups. Plasma levels of (C) thiobarbituric acid reactive species, (D) protein carbonyls, and (e) interleukin-6 in antibiotics, low interleukin-6 and high interleukin-6 antibiotics plus antioxidants groups. ATB - antibiotics; ATX - antibiotics plus antioxidants; IL - interleukin; MDA - malondialdehyde. # Different from baseline, same group; * different from the lowinterleukin- 6 group, same time. p < 0.05.

As previously described,^(11)^ NAC plus DFX increased survival in the CLP model when administered 3 hours after sepsis induction (Figure 1A). When dividing animals that received ATX using plasma IL-6 levels, it was observed that the protective effect was observed only in those animals in the high-IL-6-level group (Figure 1B). It was previously demonstrated that NAC + DFX decreased oxidative damage in this model of severe sepsis; thus, it was determined whether this protective effect was restricted to animals in the high IL-6 level group. As demonstrated in figure 1C and 1D, the antioxidant effect of NAC + DFX was similar in the two groups when antioxidants were administered 3 hours after CLP. In addition, it was determined whether antioxidant treatment presented different effects on IL-6 plasma levels. As demonstrated in figure 1E, a significant decrease in plasma IL-6 levels was observed in the high-IL-6-level group but not in the low-IL-6-level group.

**Figure 2:**
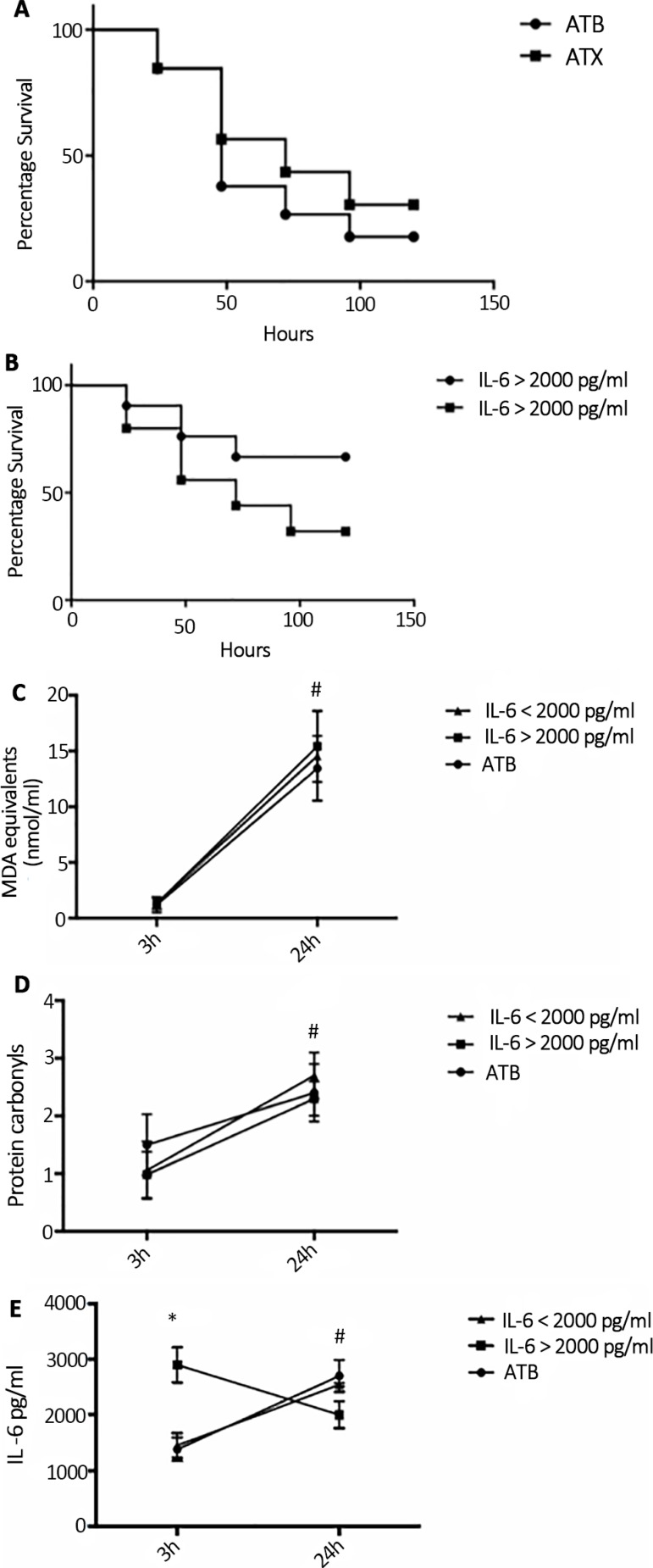
Effects of antioxidant administration twelve hours after sepsis induction in an animal model. (A) Mortality rate comparing the use of antibiotics with the use of antibiotics plus antioxidants. (B) Mortality rate comparing the use of antibiotics plus antioxidants in animals in the high- and low-interleukin- 6-level groups. Plasma levels of (C) thiobarbituric acid reactive species, (D) protein carbonyls, and (E) interleukin-6 in antibiotics, low interleukin-6 and high interleukin-6 antibiotics plus antioxidants groups. ATB - antibiotics; ATX - antibiotics plus antioxidants; IL - interleukin; MDA - malondialdehyde. # Different from baseline, same group; * different from the low-interleukin-6 group, same time. p < 0.05.

To further explore the potential of group stratification, we determined whether ATX treatment was able to increase survival when administered 12 hours after CLP. As seen in figure 2A, the protective effects of antioxidants were not significant when drugs were administered 12 hours after CLP. Despite this, a protective effect of NAC plus DFX was observed in the high-IL-6-level group even when administered 12 hours after CLP (Figure 2B). This protective effect did not seem to be related to a different antioxidant potential between the groups since both plasma TBARS and protein carbonyls did not differ when analyzed 24 hours after CLP (Figure 2C and 2D). Furthermore, there was a significant different effect on IL-6 plasma levels (Figure 2E), as demonstrated in the 3-hour group.

It was previously demonstrated that this antioxidant combination (NAC + DFX) was not able to decrease AKI incidence in critically ill patients with prolonged hypotension.^(18)^ Thus, it was decided to determine if the stratification strategy using IL-6 levels could detect patients who would benefit from this treatment. Included patients had a mean age of 51 ± 16 years, had an equal distribution of males and females, and had a mean Charlson comorbidity score of 2.6 ± 2.3, and most were admitted because of a medical condition (80%). The mean Acute Physiology and Chronic Health Evaluation (APACHE II) score was 20 ± 7, and sepsis was diagnosed in 52% of patients. As shown in table 1, patients treated with antioxidants in the high-IL-6 subgroup had a lower incidence of AKI, but not AKI severity or ICU mortality, than those in the low IL-6 subgroup (Table 1).

**Table 1:** Outcomes in patients with low and high interleukin-6 plasma levels treated with antioxidants

Outcome	Group		p value
	Low IL-6	High IL-6	
	n = 20	n = 20	
AKI incidence	16 (80)	10 (50)	0.047
AKI severity, stages 2 and 3	9 (45)	6 (30)	0.32
ICU death	14 (70)	9 (45)	0.11

IL- interleukin; AKI - acute kidney injury; ICU - intensive care unit. Results expressed as n (%).

## DISCUSSION

**Figure 3:**
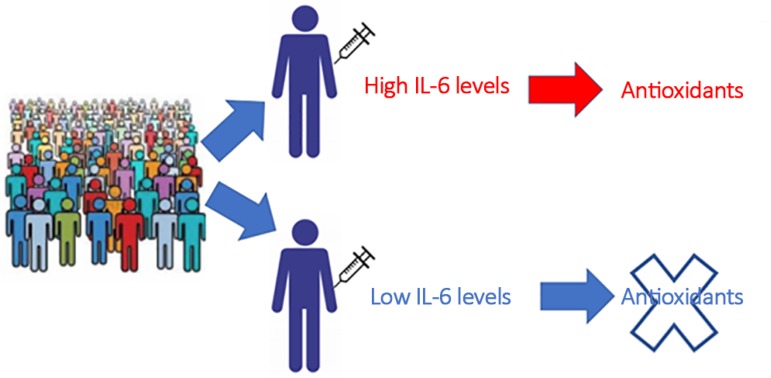
A retrospective stratification of patients by interleukin-6 plasma levels suggests beneficial effects of antioxidants for patients with high baseline circulating interleukin-6 levels. IL-6 - interleukin-6.

Here, we demonstrated that early or late administration of antioxidants improved survival in rats with high IL-6 levels, as demonstrated previously.^(7,8)^Additionally, a retrospective stratification of patients by IL-6 plasma levels suggests beneficial effects of antioxidants for patients with high baseline circulating IL-6 levels (Figure 3).

This concept is rational since the idea of a homogenous septic population is not realistic.^(24)^Beyond differences in the definition of sepsis, there is a vast array of clinical and biological combinations that can be clustered in different phenotypes that may have different outcomes and respond differently to treatment. Recently, this concept was tested in a large database, and 4 sepsis phenotypes were identified that correlated with host-response patterns and clinical outcomes.^(25)^ Additionally, it was suggested that these phenotypes may help in understanding the heterogeneity of treatment effects.^(25)^

In this context, Remick et al. demonstrated that mice at low risk of death due to sepsis had decreased survival when treated with IL-18 binding protein.^(8)^ Osuchowsk et al.^(7)^ demonstrated that early and accurate survival prediction allows targeted immunosuppression using dexamethasone, improving survival in the late phase. They proposed a new paradigm in the treatment of sepsis: the individualized evaluation of the patient’s immunoinflammatory status/risk of death followed by therapy directed only to the appropriate group.^(7)^ In clinical trials, immunomodulation with anti-TNF did not show any significant decrease in mortality,^(26)^ but if patients were stratified by baseline levels of IL-6, a reduction in mortality was reported in the patients with elevated IL-6 levels.^(9,27,28)^ This concept is also applied to other critical illnesses. Calfee et al.^(29)^ recently described that two subphenotypes of acute respiratory distress syndrome (ARDS) patients, with distinct clinical and biological features, had different clinical outcomes. One of the phenotypes, hyperinflammatory, had improved survival when treated with simvastatin.^(29)^ Here, we reinforced this concept in a translational study. These findings support further pursuit of predictive enrichment strategies in critical care clinical trials.

Furthermore, some differences between responders and nonresponders are presented here. Interestingly, there were no significant differences between these two groups in plasma oxidative damage parameters and only a slight difference in IL-6 levels. These findings are similar to the findings of Osuchowski et al and other researchers^(7,30-32)^that better survival occurred without the suppression of the typical proinflammatory mediators, such as IL-6 and IL-1ra, suggesting that the deaths were not mediated by excessive cytokine-driven inflammation.

## CONCLUSION

In conclusion, our data indicate that targeting even nonspecific therapy such as antioxidants may be beneficial when populations demonstrate a similar inflammatory phenotype.
